# A Tribute to Alessandro Moretta (1953–2018). Living Without Alessandro

**DOI:** 10.3389/fimmu.2018.01292

**Published:** 2018-06-07

**Authors:** Eric Vivier, Daniel Olive

**Affiliations:** ^1^Innate Pharma, Marseille, France; ^2^Aix Marseille Université, Marseille, France; ^3^Assistance-Publique des Hôpitaux de Marseille, Marseille, France; ^4^Institut Paoli Calmettes, Aix Marseille Université, Marseille, France

**Keywords:** NK, innate immunity, immunology, Medicine, cancer immunotherapy, immmunopathology

Alessandro Moretta was M.D. and Professor of Histology working for more than 30 years at the University of Genova (Italy) (Figure 1). For the majority of scientists, he is known as the godfather of human NK cells, although his achievements go far beyond this specific area. Many of us knew about his disease since 2010 and admired his courage during this awful period when he was still moving ahead, running his lab, and publishing outstanding articles. We were all happy that immunotherapy using anti-PD1 antibodies improved his very severe non-small cell lung carcinoma in 2014. Unfortunately, he passed away 7 February, 2018, and it is a terrible loss for the immunological community.

**Figure 1:**
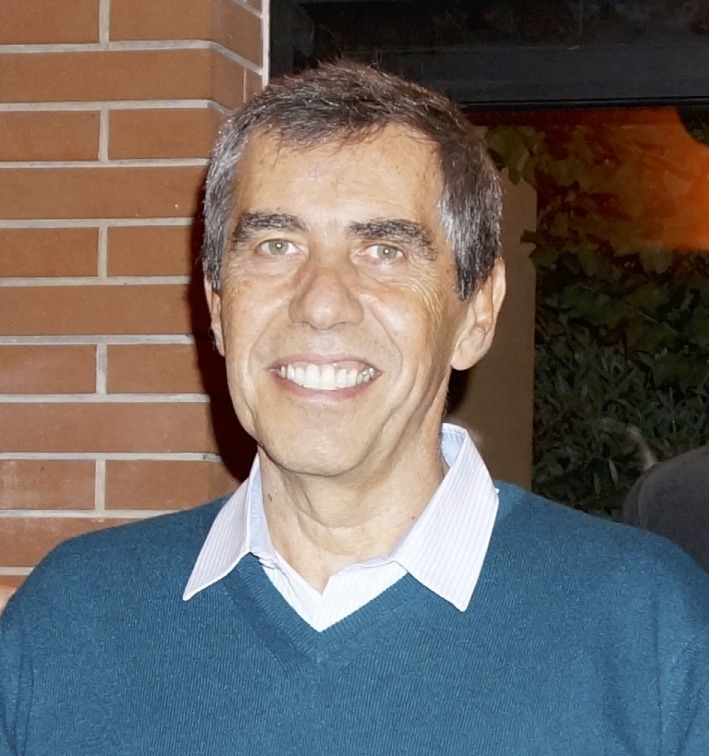
Prof. Alessandro Moretta 2014.

As a scientist, he was among the most productive colleagues with more than 500 articles and reviews since 1976. Despite the disease, he was still pursuing his objectives, namely to continue his research work as well as running a Ph.D. training program in order to promote immunology for scientists and doctors. His objective was to achieve his work and to retire at the age of 70 years.

Alessandro was both an M.D. and a scientist, and all his achievements since 1976 are along the same line: promoting high standard basic research and transferring the findings as soon as possible in patients’ studies including cancer, viral infections, and immunodeficiencies. To use the classical terms, he was from the beginning both working from bench to bedside as well as to bedside to bench. His career has included two main periods after he obtained his M.D. in 1978: Lausanne with Jean Charles Cerrotini and Lorenzo his brother, and Genova when he came back to run his lab. All the periods throughout the 42-year period were highly successful.

During the Lausanne period, he mainly focused on the definitions of human T cells populations and the identification of cytotoxic T cell (CTL). This period was highly productive and provided critical informations on cytotoxic effector cells. He was among the first ones to use efficiently the novel technologies developed in the mid-1970s following Milstein and Kohler’s discoveries, namely monoclonal antibodies (mAbs) and flow cytometers. With the help of these tools, he made an extensive delineation of cytotoxic T cells.

He also adapted T cell cloning by limiting dilution (LDA) to identify and characterize the phenotype and the function of T cell clones at the single cell level. This technology was instrumental to identify the cells possessing CTL function and cytokine production. Due to his strong translational appetite, he extended these findings to human tumor infiltrating lymphocytes as early as 1985. The discovery and a better understanding of cosignaling molecules led him to work on CD2 and Tp44 (now CD28) and their signaling capacities using the newly developed calcium flux and inositol phosphate assays. These were among the first studies investigating the signaling pathways activated and represented a first step toward pharmacological intervention. The potency of his single cell cloning technologies was extended to innate effectors namely a novel population that had just been described as γδ T cells in 1987.

Upon arrival in Genova, he dedicated his work to natural killer (NK) cells. This population had remained elusive after the initial seminal works performed in the mid-1970s both in humans and mice. He tackled this issue very efficiently as always, combining for the first time all the available technologies to understand the biology of these cells in humans. He generated mAbs with a brilliant screening strategy based on the function of NK cell clones together with his expertise in LDA. A real tour de force. A large collection of mAbs specific for these cells were generated and characterized. To identify the molecular weight and structure of the molecules recognized, he devised within his laboratory, biochemical strategies based on immunoprecipitation. The final step that was implemented in his lab was the cloning of the genes coding for the molecules that his group had identified using mAbs. Altogether, these studies created a revolution in the field of human NK cell biology by identifying inhibitory receptors, activating receptors and co-receptors.

A first category of NK receptors with inhibitory function was identified with the discovery of p58 molecules that were later called killer cell immunoglobulin-like receptors (KIRs) in collaboration with his brother, Lorenzo Moretta (1). Rapidly, he discovered that the p58 molecules were receptors specific for allotypic determinants of HLA-C molecules. He also found an homologous receptor of 50 kDa named p50 that turned out to be activating counterparts of p58 receptors, primarily differing from p50 intra-cytoplasmic domains. These discoveries produced tools that are extensively used worldwide, such as the famous EB6 and GL183 mAb, and to prepare the next step of translational investigations of immunointervention. Additional receptors interacting with HLA-class I molecules were identified during this era, namely the heterodimer made of NKG2A and CD94.

Despite being extremely appealing, the missing-self hypothesis proposed by K. Karre and H. G. Ljunggren needed to be completed as the signals activating NK cells were unknown. Again, using his NK clones and mAbs, he made multiple novel discoveries in 3 years (between 1998 and 2000) i.e., the identification of three activating receptors named natural cytotoxicity receptors (NCR): Nkp44, Nkp46, and Nkp30. He also extended these studies to co-receptors and specifically identified the functions and ligands of DNAM-1 (CD226), namely PVR and nectin-2, as well as the role of the co-receptor 2B4.

In parallel, the identification of KIRs and their recognition of HLA-class I alleles opened new avenues in the field of hematopoietic stem cell transplantation, especially in haploidentical transplantation first *via* the pioneer work of A. Velardi team in acute myeloid leukemia (AML) as soon as 1999. With a great collaboration with F. Locatelli and L. Moretta, a project was launched and currently benefits to patients with pediatric acute lymphoblastic leukemias. Alessandro devised flow cytometry-based assays to identify the donors who were more appropriate to exert this GvL effect.

In 1999, Innate Pharma was launched in Marseille. A. Moretta was enthusiastic to be part of this project and to help organizing a program dedicated to the immunomodulation of NK cells *via* the blockade of the two inhibitory receptors KIRs and NKG2A receptors. Two clinical products were generated: Lirilumab targeting KIR and Monalizumab targeting NKG2A. These two products are currently used either in combination or alone in clinical trials in cancer patients. A first class of products targeting peripheral T cell lymphomas through the KIR receptor KIR3DL2 is also currently in Phase 1 clinical trial. He developed these mAbs with Innate Pharma and M. Bagot and A. Bensussan. In addition to these immunotherapeutic interventions, his discoveries have improved the understanding of NK-target interaction in patients. He found that the NCR were down regulated in AML patients at diagnosis and that this impaired NK killing in these patients (2002). These studies were extended to NK from patients with solid tumors where he identified dysfunction of NK cells in the microenvironment (TME) of breast, ovarian, and prostate cancers. Coming back to his appetite for bed to benchside approaches, he investigated NK cells in HIV-infected patients with A. Fauci. They found profound alterations of NK cells including the accumulation of subpopulations of CD56neg/low CD16+ as well as Siglec7 deficient NK cells. Altogether, these alterations mediated by cancer and infection further supported the view that NK cells were key players in the response against these pathological conditions. Since his curiosity was inextinguishable, he also decided to explore, although he was sick, novel research areas in the field of NK cell memory and regarding the regulation of NK cell functions in the TME. These projects were ongoing as he passed away, and manuscripts were about to be submitted. We are eager to find out what his discoveries were.

On top of being an outstanding researcher, Alessandro has always been an excellent teacher. For him, the transfer of information was among the missions he wanted to achieve and friendship was instrumental. He did this at multiple meetings as well as in his lab where he has trained several generations of scientists either in Lausanne or Genova who are either independent or should become soon like G. Pantaleo, M. Lopez-Botet, G. Ferlazzo, D. Mavilio, S. Sivori, R. Castrinoci, E. Marcenaro, C. Bottino and others.

Alessandro was among the researchers who were the most appreciated and liked in the immunological community. The reasons are multiple but to sum up briefly he was always smiling and laughing, debating in a very smart and gentleman like way, and characterized by his incredible generosity in sharing information and reagents. He was so keen exchanging through congresses held in Greece that he launched with a few friends dedicated to innate immunity. The 15th edition of this meeting in June 2018 will be dedicated to honor Alessandro.

We have to give a special message to those who were with him during his last years including his wife Genny, and his brother Lorenzo who was always taking care of him, as well as his lab that was fully dedicated to him.

In addition to science, Alessandro was addicted to music (although not playing anymore guitar) and especially the songs of the 1970s. So, let us finish with “when the music is over” we have to go the “stairway to heaven” and his favorites, the famous Rolling Stones song “it’s only rock and roll and I like it” translated by Alessandro to “it’s only innate immunity and I like it” (2).

Ciao bello, noi manchi già.

## Author Contributions

EV and DO have equally contributed to this opinion regarding Prof. Alessandro Moretta.

## Conflict of Interest Statement

The authors declare that the research was conducted in the absence of any commercial or financial relationships that could be construed as a potential conflict of interest.

## References

[1] Moretta A1 Tambussi G Bottino C Tripodi G Merli A Ciccone E (1990). A novel surface antigen expressed by a subset of human CD3− CD16+natural killer cells. Role in cell activation and regulation of cytolytic function. J Exp Med. 171(3):695–714. 2137855 10.1084/jem.171.3.695 PMC2187781

[2] Marcenaro E1 Della Chiesa M Dondero A Ferranti B Moretta A (2007). It’s only innate immunity but I like it. Adv Exp Med Biol. 590:89–101. 17191379 10.1007/978-0-387-34814-8_6

